# 

**Published:** 1878-12

**Authors:** 


### Table of Contents.

ORIGINAL COMMUNICATIONS.

American Pharmaceutical Association — Annual Address of President Saunders .... 513

Diphtheria Analogous to Erysipellatous Inflammation. *By W. O'Daniel, M.D., Bullard's, Ga.* .... 530

Diphtheria. *By H. C. Ryals, M.D., McVille, Ga.* .... 532

REPORTS OF SOCIETIES.

Atlanta Academy of Medicine .... 534

SELECTIONS.

Procidentia Uteri and its Treatment .... 537

Recent Progress in Pathology and Pathological Anatomy .... 543

Gastric Ulcer .... 547

Unusual Effects of Quinine .... 554

EDITORIAL.

Yellow Fever Commission .... 558

Meteorological Report for November .... 566

CORRESPONDENCE.

European Correspondence .... 567

BIBLIOGRAPHICAL.

On Rest and Pain .... 571

On the Treatment of the Various Forms of Acne and of Rosacea .... 571

Essentials of Chemistry, Inorganic and Organic .... 571

EXCERPTA.

The Man-Fish of Tennessee .... 572

Transfusion .... 574

The Eucalyptus in Malarial Disorders .... 575

How to Kill a Tapeworm in an Hour .... 576

Tansy in Pruritis Vulvæ .... 576

Epistaxis Arrested by Subcutaneous Injections of Ergotine .... 576

